# Rab5 Enhances Classical Swine Fever Virus Proliferation and Interacts with Viral NS4B Protein to Facilitate Formation of NS4B Related Complex

**DOI:** 10.3389/fmicb.2017.01468

**Published:** 2017-08-08

**Authors:** Jihui Lin, Chengbao Wang, Longxiang Zhang, Tao Wang, Jing Zhang, Wulong Liang, Cheng Li, Gui Qian, Yueling Ouyang, Kangkang Guo, Yanming Zhang

**Affiliations:** College of Veterinary Medicine, Northwest A&F University Yangling, China

**Keywords:** classical swine fever virus, NS4B, Rab5, interaction, NS4B related complex

## Abstract

Classical swine fever virus (CSFV) is a fatal pig pestivirus and causes serious financial losses to the pig industry. CSFV NS4B protein is one of the most important viral replicase proteins. Rab5, a member of the small Rab GTPase family, is involved in infection and replication of numerous viruses including hepatitis C virus and dengue virus. Until now, the effects of Rab5 on the proliferation of CSFV are poorly defined. In the present study, we showed that Rab5 could enhance CSFV proliferation by utilizing lentivirus-mediated constitutive overexpression and eukaryotic plasmid transient overexpression approaches. On the other hand, lentivirus-mediated short hairpin RNA knockdown of Rab5 dramatically inhibited virus production. Co-immunoprecipitation, glutathione S-transferase pulldown and laser confocal microscopy assays further confirmed the interaction between Rab5 and CSFV NS4B protein. In addition, intracellular distribution of NS4B-Red presented many granular fluorescent signals (GFS) in CSFV infected PK-15 cells. Inhibition of basal Rab5 function with Rab5 dominant negative mutant Rab5S34N resulted in disruption of the GFS. These results indicate that Rab5 plays a critical role in facilitating the formation of the NS4B related complexes. Furthermore, it was observed that NS4B co-localized with viral NS3 and NS5A proteins in the cytoplasm, suggesting that NS3 and NS5A might be components of the NS4B related complex. Taken together, these results demonstrate that Rab5 positively modulates CSFV propagation and interacts with NS4B protein to facilitate the NS4B related complexes formation.

## Introduction

Classical swine fever (CSF), caused by the classical swine fever virus (CSFV), is one of the economically important infectious diseases in the global swine industry. CSFV belongs to the genus *Pestivirus* within the family *Flavivridae*. The genome of CSFV is a linear, non-segmented, single stranded and positive-sense RNA with 12.3 kb in size (Moennig, 2015; Rossi et al., 2015). The virus genome carries one large open reading frame (ORF) flanked by 5′ and 3′ untranslated regions (Meyers et al., 1989). The ORF encodes a precursor polypeptide which is co- and post-translationally cleaved into four structural proteins (C, E^rns^, E1 and E2) and eight non-structural proteins (N^pro^, p7, NS2, NS3, NS4A, NS4B, NS5A and NS5B) (Lin et al., 2014). Viral replicase proteins NS3-NS5B accompanied by 5′ and 3′ untranslated regions are the minimal viral elements required by CSFV RNA replication (Behrens et al., 1998).

Like other members of the *Flaviviridae*, such as hepatitis C virus (HCV) and bovine viral diarrhea virus (BVDV), CSFV NS4B protein is an intracellular membrane-associated viral replicase protein (Hugle et al., 2001; Weiskircher et al., 2009). It has been shown that HCV NS4B specifically binds to the 3′ terminus of the negative strand of the viral genome, which implies that NS4B tethers HCV RNA onto the replication complexes and facilitates positive sense RNA synthesis (Einav et al., 2008). BVDV NS4B was shown to interact with NS3 and NS5A, which are involved in viral RNA synthesis and modulates virus cytopathogenicity (Qu et al., 2001). CSFV NS4B is also essential for virus lifecycle in host. Mutation of the putative Toll/interleukin-1 receptor (TIR) -like domain in the C-terminal region of NS4B leads to an attenuated phenotype and reduces replication of highly virulent Brescia strain (Fernandez-Sainz et al., 2010). Modulation of NS4B NTPase activity results in no infectious virus or virus replication capability impairment, and the recovered mutant viruses reserve a virulent phenotype as parental strain Brescia (Gladue et al., 2011). Furthermore, a chimeric low virulent GPE^-^ derived virus carrying the complete Eystrup NS4B presents significant higher replication efficiency (Tamura et al., 2012). Until now, most of studies have been focusing on the roles of the CSFV NS4B protein in viral genomic replication or virulence (Fernandez-Sainz et al., 2010; Tamura et al., 2012, 2015), but knowledge on the interaction of NS4B with host proteins and their impacts on CSFV replication is limited.

The small GTPase Rab5, known as marker protein of early endosome, cycles between active (GTP-bound) and inactive (GDP-bound) forms (Vitale et al., 1995) to regulate various cellular functions, such as endocytosis, vesicular transport and early endosome fusion (Grosshans et al., 2006; Su et al., 2011). Many studies are focusing on the roles of Rab5 in virus uptake and replication. Rab5 is implicated in productive infection of Venezuelan equine encephalitis virus, avian reovirus and Epstein-Barr virus (Colpitts et al., 2007; Huang et al., 2011; Watanabe et al., 2017). The observations that Rab5 co-localizes with yellow head virus particles and with bursal disease virus VP2 protein suggest that Rab5 is involved in these two viruses’ intracellular delivery (Gimenez et al., 2015; Posiri et al., 2016). Rab5 is also involved in the relocalization of HIV-1 from raft-rich region to early endosomes and participates in the movement of influenza virus from the cell periphery to the perinuclear region (Vidricaire and Tremblay, 2005; Liu et al., 2014). Overexpression of wild type Rab5 (Rab5WT) or active form Rab5 (Rab5Q79L) can enhance adenovirus and bovine ephemeral fever virus infection (Rauma et al., 1999; Cheng et al., 2012). On the other hand, silencing Rab5 or expressing the dominant negative mutant Rab5S34N significantly decreases foot-and-mouth disease virus infection (Johns et al., 2009). *Acanthamoeba polyphaga* mimivirus even encodes a putative Rab5 homolog to recruit vps34, Atg-8 and dynamin for virus replication and particle assembly (Zade et al., 2015).

Rab5 is required for cell entry of dengue and West Nile viruses, as well as essential for their survival in host cells (Krishnan et al., 2007; Acosta et al., 2012; Ayala-Nunez et al., 2016). Rab5 is one of the components of HCV NS4B-bound replication complexes, and inhibition of Rab5 function leads to significant decrease of HCV replication (Stone et al., 2007; Manna et al., 2010). Recently, Rab5 has been shown to co-localize with CSFV particles and to be required for virus infection (Shi et al., 2016). To date, effects of Rab5 on CSFV replication remains unknown. In this study, we identified for the first time Rab5 as a factor that promotes CSFV proliferation and showed that Rab5 interacts with viral NS4B protein to facilitate formation of NS4B related complexes.

## Materials and Methods

### Cells, Virus and Antibodies

Porcine kidney (PK-15) cells (ATCC: CCL-33) were cultured in high glucose Dulbecco’s-modified Eagle’s medium (DMEM; Gibco, United Kingdom) supplemented with 10% Fetal Bovine Serum (FBS; Gibco, United Kingdom), 100 U/mL penicillin and 100 μg/mL streptomycin at 37°C under a 5% CO_2_ incubator. Human embryonic kidney (HEK-293T) cells (ATCC: CRL-11268) grown in high glucose DMEM containing 10% FBS, 1.5 g/L sodium bicarbonate, 4.5 g/L glucose, 0.1 mM non-essential amino acids, 1.0 mM sodium pyruvate, 2 mM L-glutamine and 10 mM HEPES. CSFV Shimen strain (GenBank: AF092448) was obtained from the China Institute of Veterinary Drug Control (Beijing, China) and was propagated in PK-15 cells.

Anti-Rab5 rabbit polyclonal antibody (sc-309) was purchased from Santa Cruz Biotechnology. Anti-E2 antibody was produced in mouse (Ab-mart, Shanghai, China). Anti-GST tag (CW0085S) and anti-c-Myc tag (CW00899M) rabbit polyclonal antibodies, anti-Flag tag (CW0287M) and anti-GAPDH (CW0101M) mouse monoclonal antibodies were purchased from Beijing CWBIO. HRP-labeled Goat Anti-Mouse (A0216) and Anti-Rabbit (A0208) IgG were produced at Beyotime Biotechnology (Shanghai, China). Anti-c-Myc Agarose Affinity Gel antibody produced in rabbit (SIGMA-ALDRICHs, A7470) and anti-flag M2 Affinity Gel antibody produced in mouse (SIGMA-ALDRICHs, A2220) were used.

### Plasmids and Transfection

CSFV NS4B encoding sequence was amplified by RT-PCR and inserted into the pDsRed-N1 and pcDNA3.1 (++) vectors (Clontech) to generate plasmids pFlagNS4B-Red and pcDNA-NS4B-Flag, respectively. In pFlagNS4B-Red, NS4B was separately flanked by flag tag and DsRed at its N-terminus and C-terminus. In pcDNA-NS4B-Flag, NS4B was fused by flag tag at its C-terminus. CSFV NS3 and NS5A encoding sequences were separately cloned into pEGFP-N1 (Clontech) to get pNS3-GFP and pNS5A-GFP plasmids. Swine Rab5 (GenBank: NM_001123180) cDNA was inserted into pEGFP-N1, pcDNA3.1 (+) and pGEX-6P-1 (#28-9546-48; GE Healthcare) vectors to generate pRab5-GFP, pcDNA-Rab5-Myc and pGEX-GST-Rab5, respectively. The pGEX-GST-Rab5 was transformed into *E. coli* BL21 (DE3) (Invitrogen, Carlsbad, CA, United States) to produce fused protein. Plasmid pRab5S34N-GFP expressing Rab5 dominant negative mutant Rab5S34N, which contained a substitution mutation at residue 34 from serine to asparagine in the first GTP/GDP binding motif, was constructed as described previously (Li and Stahl, 1993; Sakurai et al., 2010) using the TaKaRa MutanBEST Kit (Takara Bio, Dalian, China, #R401) and mutagenesis primers (S34N-F and S34N-R). Swine Rab2 (GenBank: XM_001926704) and CSFV NS5A cDNA were separately cloned into pcDNA3.1 (+) to generate pcDNA-NS5A-Flag and pcDNA-Rab2-Myc. All primers and restriction enzymes used for plasmids construction are shown in **Table 1**. All eukaryotic expression plasmids were transfected into target cells using the TurboFect Transfection Reagent (Thermo scientific, #R0531).

**Table 1:** Primers and restriction enzymes used for plasmids construction.

Primer	Sequence (5′-3′)	Restriction Enzyme	Use
NS4B-Flag-F	CCCAAGCTTGCCACCATGGCTCAGGGGGATGTGCAGAGA	Hind III	Construction of pcDNA-NS4B-Flag
NS4B-Flag-R	CGCGGATCCTTA**CTTATCGTCGTCATCCTTGTAATC**TAGCTGGCGGATCTTTCCTTCA	BamH I	
NS5A-Flag-F	CCCAAGCTTGCCACCATGTCAAGTAATTACATTCTAGAGCTCCT	Hind III	Construction of pcDNA-NS5A-Flag
NS5A-Flag-R	CGCGGATCCTTA**CTTATCGTCGTCATCCTTGTAATC**CAGTTTCATAGAATACACTTTTGC	BamH I	
Rab5-Myc-F	CCCAAGCTTGCCACCATGGCTAATCGAGGAGCAAC	Hind III	Construction of pcDNA-Rab5-Myc
Rab5-Myc-R	CGCGGATCCTTA**CAGATCCTCTTCAGAGATGAGTTTCTGCTC**ATTACTACAACACTGACTCCTGGT	BamH I	
Rab2-Myc-F	CTAGCTAGCGCCACCATGGCGTACGCCTATCTCTTCAAG	Nhe I	Construction of pcDNA-Rab2-Myc
Rab2-Myc-R	CGCGGATCCTCA**CAGATCCTCTTCAGAGATGAGTTTCTGCTC**ACAGCAGCCTCCGCCGG	BamH I	
NS4B-Red-F	CCCAAGCTTATG**GATTACAAGGATGACGACGATAAG**GCTCAGGGGGATGTGCAGAGA	Hind III	Construction of pNS4B-Red
NS4B-Red-R	CGCGGATCCCGTAGCTGGCGGATCTTTCCTTCA	BamH I	
NS3-GFP-F	CCCAAGCTTATGGGGCCTGCCGTTTGCA	Hind III	Construction of pNS3-GFP
NS3-GFP-R	CGCGGATCCCGTAGACCAACTACTTGTTTTAGTGCTC	BamH I	
NS5A-GFP-F	CCCAAGCTTATGTCAAGTAATTACATTCTAGAGCTCCT	Hind III	Construction of pNS5A-GFP
NS5A-GFP-R	CGCGGATCCCGCAGTTTCATAGAATACACTTTTGC	BamH I	
Rab5-GFP-F	CCCAAGCTTATGGCTAATCGAGGAGCAAC	Hind III	Construction of pRab5-GFP
Rab5-GFP-R	CGCGGATCCCGATTACTACAACACTGACTCCTGGT	BamH I	
S34N-F	GGCAAAAATAGCCTAGTGCTTCGTTTT		Construction of pRab5S34N-GFP
S34N-R	TAGGCTATTTTTGCCAACAGCAGACTC		
Rab5-LV-F	CGGAATTCATGGCTAATCGAGGAGCAAC	EcoR I	Construction of pCDH-LV-Rab5
Rab5-LV-R	CGGGATCCATTACTACAACACTGACTCCTGGT	BamH I	
GST-Rab5-F	CGGGATCCATGGCTAATCGAGGAGCAAC	BamH I	Construction of pGEX-GST-Rab5
GST-Rab5-R	CCGCTCGAGATTACTACAACACTGACTCCTGGT	Xho I	

*Underlines show restriction enzyme sites or mutagenesis sites, bolds show flag or c-myc tag*.

### Construction of Stable Cell Lines with Rab5 Overexpression or Knockdown

Recombinant lentiviruses overexpressing Rab5 or with the gene knockdown were prepared as previously described (Zhang et al., 2015). Briefly, the Rab5 cDNA was cloned into over-expression lentivector pCDH-CMV-MCS-EF1-GreenPuro (SBI, Mountain View, CA, United States) to obtain pCDH-LV-Rab5. Three pairs of short hairpin RNA (shRNA) targeting swine Rab5 and a scrambled non-targeting shRNA (see **Table 2**) were inserted into pCDH-U6-MCS-EF1-GreenPuro (SBI, Mountain View, CA, United States) to obtain pCDH-U6-Rab5-sh1, pCDH-U6-Rab5-sh2, pCDH-U6-Rab5-sh3 and pCDH-U6-Rab5-shN (negative control), respectively. Each construct along with three assistant plasmids (pGAG, pREV and pVSV-G, 3:1:1:1) were transfected into HEK-293T cells at logarithmic phase. The supernatants were collected as lentiviruses stocks 48 h post transfection. The Rab5 overexpression or shRNA recombinant lentiviruses (MOI = 1) were used to infect PK-15 cells at a confluence of 70% in a 12-well plate after removing half of the complete medium. Fresh complete medium was used to refresh the medium 12 h post infection (hpi), and puromycin (SIGMA-ALDRICHs, P8833) was then added (5 μg/mL) to select positive cells.

**Table 2:** Short hairpin RNA (shRNA) sequences.

shRNA	Sequence (5′-3′)
Rab5-sh1-S	GATCCGGGCCAATTTCATGAATTTCACAAGAGTGAAATTCATGAAATTGGCCCTTTTTG
Rab5 -sh1-A	AATTCAAAAAGGGCCAATTTCATGAATTTCACTCTTGTGAAATTCATGAAATTGGCCCG
Rab5 -sh2-S	GATCCGCCTAGCACCAATGTACTACACAAGAGTGTAGTACATTGGTGCTAGGCTTTTTG
Rab5 -sh2-A	AATTCAAAAAGCCTAGCACCAATGTACTACACTCTTGTGTAGTACATTGGTGCTAGGCG
Rab5 -sh3-S	GATCCGCACAGTCCTATGCAGATGACCAAGAGGACATCTGCATAGGACTGTGCTTTTTG
Rab5 -sh3-A	AATTCAAAAAGCACAGTCCTATGCAGATGACCTCTTGGACATCTGCATAGGACTGTGCG
Rab5- shN-S	GATCCGCTTAAACGCATAGTAGGACTCAAGAGAGTCCTACTATGCGTTTAAGCTTTTTG
Rab5 -shN-A	AATTCAAAAAGCTTAAACGCATAGTAGGACTCTCTTGAGTCCTACTATGCGTTTAAGCG

*Underlines show loop ring*.

### RNA Extraction and Real-Time RT-PCR

Quantitative real-time RT-PCR (qPCR) was conducted to detect changes in Rab5 mRNA and CSFV RNA levels using primers listed in **Table 3**. RNAiso Plus (Takara Bio, Dalian, China, #9108) was used to extract total cellular RNA, and cDNA was obtained by reverse transcription reactions using PrimeScript RT reagent Kit (Takara Bio, Dalian, China, #RR047A). The qPCR reactions were carried out in 96-well blocks with an iCycler iQ5 RealTime Detection System (Bio-Rad, Hercules, CA, United States) using SYBR ExScript^TM^ RT-PCR Kit (Takara Bio, Dalian, China, #RR820A), and the reaction conditions were as follows: 95°C for 30 s, then 40 cycles at 95°C for 5 s and 60°C for 30 s. All qPCR reactions were carried out in technical and biological triplicate. The housekeeping gene *gapdh* was used as reference gene to normalize expression levels of the target gene, and the relative gene expression was calculated by using the 2^-ΔΔCt^ method (Livak and Schmittgen, 2001).

**Table 3:** Primers used for qPCR analysis.

Primer	Sequence (5′-3′)	Use
qCSFV-F	GATCCTCATACTGCCCACTTAC	qPCR detection of CSFV RNA
qCSFV-R	GTATACCCCTTCACCAGCTTG	
qgapdh-F	TTTGTGATGGGCGTGAACC	qPCR detection of GAPDH mRNA
qgapdh-R	CAGTCTTCTGGGTGGCAGTGAT	
qRab5-F	GGAGAGTCTGCTGTTGGCAAA	qPCR detection of Rab5 mRNA
qRab5-R	GGTGCTAGGCTATGGTATCGTTC	

### Western Blots

Cells cultured in 6-well plates were rinsed with PBS buffer, harvested into centrifuge tubes then resuspended in western blot or IP lysis buffer with the proteinase inhibitor PMSF (Beyotime Biotechnology, #ST506) and incubated on ice for 45 min. Concentration of proteins was determined using a BCA Protein assay Kit (Beyotime Biotechnology, #P0012S). Same amounts of proteins were loaded and separated by 12% SDS-PAGE, and then transferred onto 0.22-μm PVDF membranes (Millipore, Billerica, MA, United States). Membranes were blocked with 5% skim milk at room temperature for 2 h and then incubated with 2% blocking solution containing the primary antibodies at the dilution suggested by the manufacturer at 4°C overnight. The membranes were then washed three times with TBST and incubated with the secondary HRP-conjugated antibodies diluted at 1: 5000 in 2% blocking solution for 2 h at room temperature. After washed another three times, the immunoreactive bands were detected by using chemiluminescent detection reagent WesternBright ECL (Advansta, United States, #R-03026-D100) and analyzed by Tanon-410 automatic gel imaging system (Shanghai Tianneng, China).

### Co-immunoprecipitation (co-IP) Assays

Plasmid pcDNA-NS4B-Flag was transfected alone or co-transfected with pcDNA-Rab5-Myc into PK-15 cells. Plasmids pcDNA-NS5A-Flag and pcDNA-Rab5-Myc as well as pcDNA-NS4B-Flag and pcDNA-Rab2-Myc were co-transfected in to PK-15 cells and served as negative control. After 48 h post transfection, the cells were resuspended in western blot and IP lysis buffer with PMSF, and the lysate was used for co-IP assays utilizing ANTI-FLAG M2 Affinity Gel or Anti-c-Myc Agarose Affinity Gel antibody according to the manufacturers’ protocol. Briefly, resin (50 μL) was centrifuged at 8,000 *g* for 30 s to remove the stock solution and washed twice with TBS (50 mM Tris-HCl, 150 mM NaCl, pH 7.4). Then, 1 mL of cell lysate was added to the rinsed resin and rocked gently overnight at 4°C. Thereafter, the resin was washed three times with TBS, and the agarose beads pellet was resuspended in 2 × SDS loading buffer followed by boiling for 10 min. Finally, SDS-PAGE and immunoblotting were conducted to detect the supernatant.

### GST-Pulldown Assays

Plasmids pGEX-6p-1 and pGEX-GST-Rab5 were transformed into *E. coli* BL21 (DE3) (Invitrogen, Carlsbad, CA, United States) to express GST and GST-Rab5 proteins, respectively. Glutathione agarose resin (Thermo Scientific, #21516) balanced with equilibrium liquid (TBS and Pull-Down lysis buffer at a ratio of 1:1) was utilized to combine the proteins into complexes according to the manufacturers’ direction. Then, the complexes were incubated with NS4B-Flag expressed by HEK-293T cells transfected with plasmid pcDNA-NS4B-Flag at 4°C for 2 h. After that, elution buffer (1 mL TBS containing 3.1 g glutathione) was used to separate the combined proteins and the eluted proteins were analyzed by western blot.

### Confocal Microscopy

PK-15 cells were seeded on glass coverslips in cell culture dishes of 35 mm diameter and cultured overnight. Then, PK-15 cells were transfected with pFlagNS4B-Red or co-transfected with several other plasmids (pNS3-GFP, pNS5A-GFP and pRab5-GFP, respectively). The cells were further cultured for 48 h, rinsed with pre-cooled PBS, fixed with 4% paraformaldehyde for 10 min at room temperature and incubated with DAPI at 37°C for 20 min. Images were captured by laser scanning confocal microscopy (LSM510 META; Zeiss, Germany).

### Statistical Analysis

All data are presented as means ± standard deviations (SD). Student’s *t-*test was used to evaluate the differences between each group and a *P*-value < 0.05 was considered to be statistically significant. All statistical analyses and calculations were performed using GraphPad Prism 5 (GraphPad Software Inc., La Jolla, CA, United States).

## Results

### Upregulation of Rab5 Expression Promotes CSFV Propagation

**FIGURE 1:**
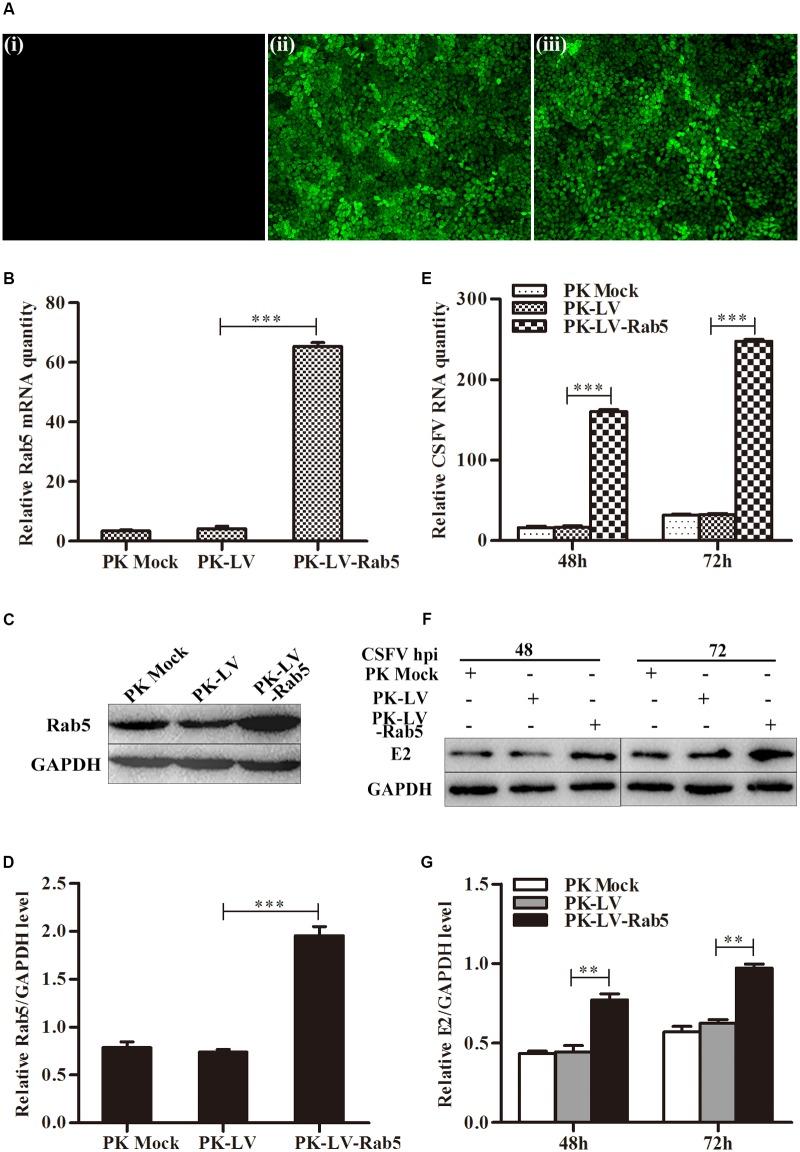
Stable overexpression of Rab5 promotes CSFV propagation. **(A)** GFP visualization of PK-15 cell lines stably overexpressing Rab5 (100 × ). (i) Mock, negative control group without recombinant lentiviruses infected PK-15 cells. (ii) Negative control group with cells infected with GFP expressing recombinant lentiviruses. (iii) PK-15 cells infected with Rab5 and GFP co-expressing recombinant lentiviruses. **(B–D)** qPCR and western blot analyses of overexpression of Rab5 in PK-15 cell lines. **(B)** qPCR analyzes Rab5 mRNA level in PK-15 cell lines with Rab5 overexpression. **(C,D)** Western blot analyzes Rab5 protein level in PK-15 cell lines with Rab5 overexpression. **(E–G)** Effects of Rab5 overexpression on CSFV propagation. **(E)** qPCR analyzes CSFV RNA level in PK-15 cell lines with Rab5 overexpression. **(F,G)** Western blot analyzes CSFV E2 protein level in PK-15 cell lines with Rab5 overexpression. Rab5 mRNA and CSFV RNA levels were analyzed by qPCR and normalized with GAPDH mRNA level. Rab5 and E2 proteins were analyzed by western blot and densitometry, and the intensity of the signal for targeted protein were normalized to that from GAPDH. Results are shown as the mean ± SD (*n* = 3). ^∗^*P* < 0.05; ^∗∗^*P* < 0.01; ^∗∗∗^*P* < 0.001; ns, no significant (*P* > 0.05).

To investigate whether Rab5 is involved in CSFV propagation, PK-15 cells overexpressing Rab5 (PK-LV-Rab5) or GFP (PK-LV) were constructed using recombinant lentiviruses. The cells were infected with CSFV at an MOI of 0.1. Fluorescence assays showed that the reporter GFP was expressed within both PK-LV-Rab5 and PK-LV cells (**Figure 1A**). The Rab5 expression was increased both at the gene and protein levels in PK-LV-Rab5 cells compared to those in PK-LV cells (**Figures 1B–D**). Along with the increase of Rab5 expression, CSFV RNA and E2 protein expression in PK-LV-Rab5 cells were enhanced significantly at 48 and 72 hpi, compared to the expression levels in PK-LV cells (**Figures 1E–G**).

**FIGURE 2:**
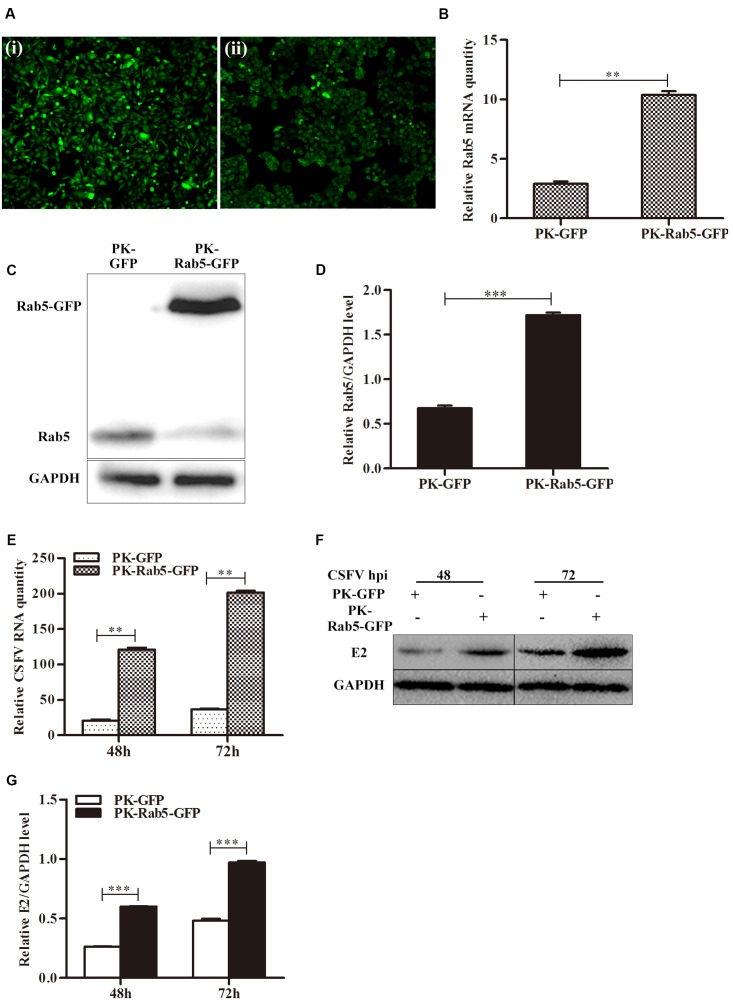
Transient overexpression of GFP-fused Rab5 promotes CSFV proliferation. **(A)** GFP visualization of PK-15 cells transiently overexpressing Rab5 (100 × ). (i) Negative control PK-15 cells transfected with pEGFP-N1. (ii) PK-15 cells transfected with pRab5-GFP. **(B–D)** qPCR and western blot analyses of Rab5 overexpression in pRab5-GFP transfected PK-15 cells. **(B)** qPCR analyzes Rab5 mRNA level in transfected PK-15 cells. **(C,D)** Western blot analyzes Rab5 protein level in transfected PK-15 cells. **(E–G)** Effects of transient Rab5 overexpression on CSFV proliferation. **(E)** qPCR analyzes CSFV RNA level in transfected PK-15 cells. **(F,G)** Western blot analyzes CSFV E2 protein level in transfected PK-15 cells. Rab5 mRNA and CSFV RNA levels were analyzed by qPCR and normalized with GAPDH mRNA level. Rab5 and E2 proteins were analyzed by western blot and densitometry, and the intensity of the signal for targeted protein were normalized to that from GAPDH. Results are shown as the mean ± SD (*n* = 3). ^∗^*P* < 0.05; ^∗∗^*P* < 0.01; ^∗∗∗^*P* < 0.001; ns, no significant (*P* > 0.05).

Additionally, PK-15 cells were transfected with pRab5-GFP (PK-Rab5-GFP) or pEGFP-N1 (PK-GFP) and infected with CSFV at an MOI of 0.1 at 24 h post transfection. Consistent with the results mentioned above, transient overexpression of Rab5 also could significantly promote CSFV propagation (**Figure 2**). It was observed that Rab5 was overexpressed with a fusion GFP (**Figures 2A–D**), and that levels of CSFV RNA and E2 protein were increased (**Figures 2E–G**). These results indicate that Rab5 promotes CSFV propagation.

### Knockdown of Rab5 Suppresses CSFV Proliferation

**FIGURE 3:**
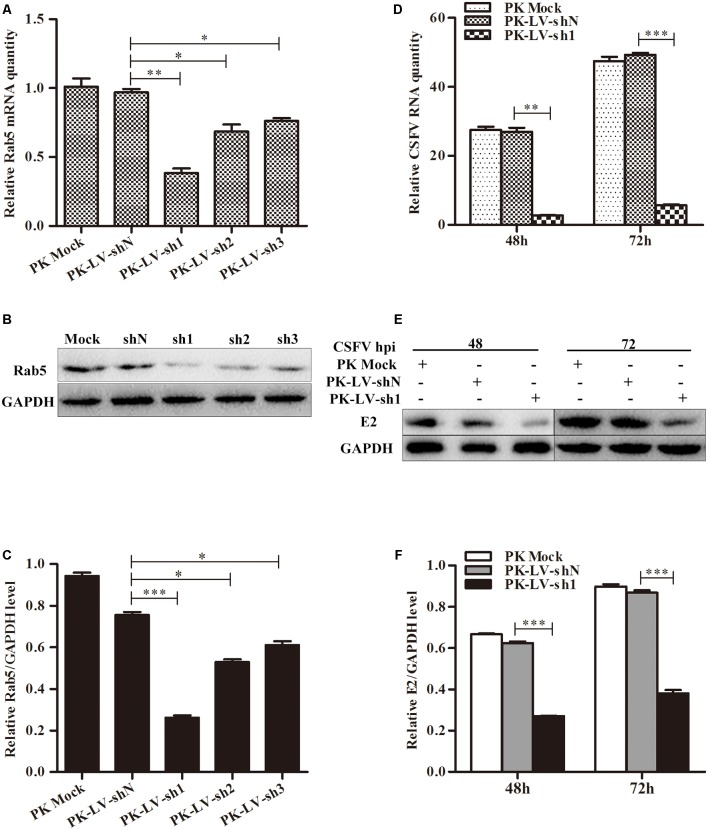
Knockdown of Rab5 decreases CSFV propagation. **(A–C)** Knockdown of Rab5 in PK-15 cells by lentivirus-mediated shRNA interference. **(A)** qPCR analyzes Rab5 mRNA level in PK-15 cell lines with Rab5 knockdown. **(B,C)** Western blot analyzes Rab5 protein level in PK-15 cell lines with Rab5 knockdown. **(D–F)** Analysis of CSFV growth rates in Rab5 knockdown and control PK-15 cell lines. **(D)** qPCR analyzes CSFV RNA level in PK-15 cell line with Rab5 knockdown. **(E,F)** Western blot analyzes CSFV E2 protein level in PK-15 cell lines with Rab5 knockdown. Rab5 mRNA and CSFV RNA levels were analyzed by qPCR and normalized with GAPDH mRNA level. Rab5 and E2 proteins were analyzed by western blot and densitometry, and the intensity of the signal for targeted protein were normalized to that from GAPDH. Results are shown as the mean ± SD (*n* = 3). ^∗^*P* < 0.05; ^∗∗^*P* < 0.01; ^∗∗∗^*P* < 0.001; ns, no significant (*P* > 0.05).

To further examine the effects of Rab5 on CSFV proliferation, recombinant lentiviruses expressing three different shRNAs (sh1, sh2, and sh3) against Rab5 and a non-targeting shN were separately transduced into PK-15 cells. PK-LV-sh1 cells were shown to have the highest efficiency at inactivating Rab5 both at the gene and protein levels (**Figures 3A–C**). Then, cell lines (PK-LV-sh1 and PK-LV-shN) and PK-15 cells were infected with CSFV at an MOI of 0.1. The results showed that knockdown of Rab5 led to significant reduction of virus RNA and E2 protein in PK-LV-sh1 cells, compared to the control cells both at 48 and 72 hpi (**Figures 3D–F**). These results suggested that knockdown of Rab5 had an inhibitory effect on CSFV production.

### CSFV Propagation Does Not Influence Rab5 Expression in PK-15 Cells

**FIGURE 4:**
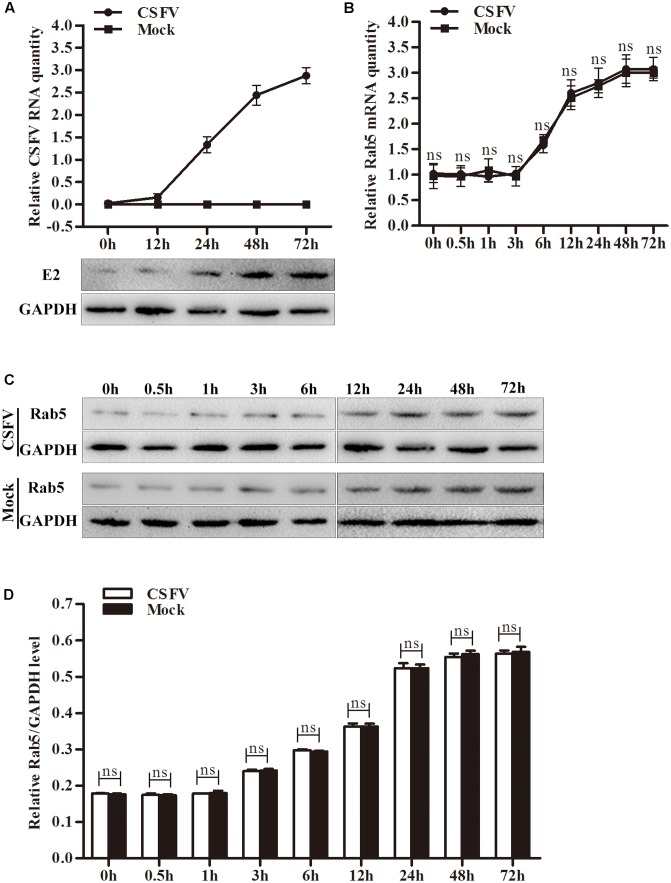
Classical swine fever virus (CSFV) replication does not change Rab5 expression. **(A)** qPCR and western blot assessment of CSFV propagation in PK-15 cells infected or mock infected with CSFV. **(B)** qPCR measurement of Rab5 mRNA level between CSFV infected and mock infected PK-15 cells. **(C,D)** Western blot and densitometry analysis of Rab5 protein levels in PK-15 cells infected or mock infected with CSFV. Results are shown as the mean ± SD (*n* = 3). ^∗^*P* < 0.05; ^∗∗^*P* < 0.01; ^∗∗∗^*P* < 0.001; ns, no significant (*P* > 0.05).

To check whether CSFV propagation would affect Rab5 expression, PK-15 cells were infected with CSFV at an MOI of 0.1. Rab5 expression levels were evaluated at various time points including the early infection stage and later proliferation processes of CSFV. As shown in **Figures 4B–D**, Rab5 expression did not change compared to the one in mock cells either at the stage of entry or at the propagation level in cells infected with CSFV.

### CSFV NS4B Binds to Rab5

**FIGURE 5:**
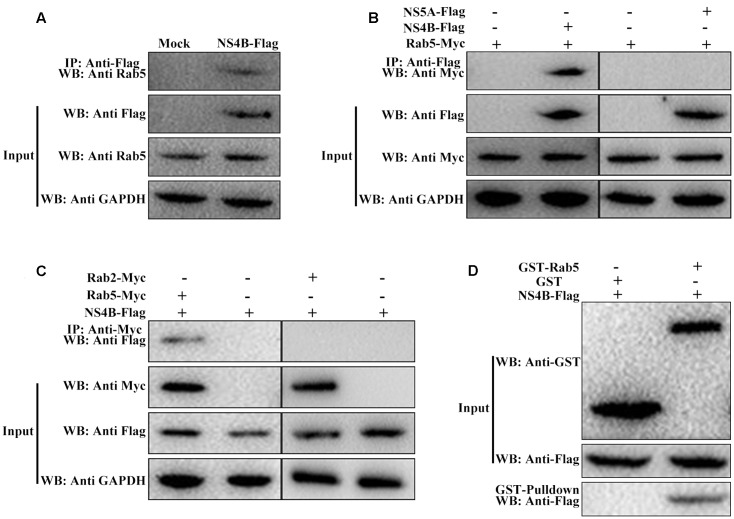
CSFV NS4B protein interacts with Rab5. **(A–C)** Co-immunoprecipitation (co-IP) analyses of the interaction between NS4B and Rab5. **(A)** Endogenous Rab5 bound to NS4B-Flag in PK-15 cells. PK-15 cells were transfected or mock transfected with pcDNA-NS4B-Flag for 48 h. The transfected or untransfected cells were lysed and immunoprecipitated with antibody against Flag followed by western blot analysis using the anti-Flag monoclonal antibody (MAb) (1:1000) and a rabbit anti-Rab5 polyclonal antibody (PAb) (1:200). **(B)** Exogenous Rab5-Myc bound to NS4B-Flag in co-transfected PK-15 cells. Cell lysates from PK-15 cells transfected with pcDNA-NS4B-Flag alone or co-transfected with pcDNA-NS4B-Flag and pcDNA-Rab5-Myc were immunoprecipitated with antibody against Flag followed by western blot analysis using anti-Flag MAb (1:1000) and anti-Myc PAb (1:500), NS5A-Flag and Rab5-Myc served as negative control. **(C)** Reciprocal co-IP experiments showed that exogenous Rab5-Myc precipitated NS4B-Flag, Rab2-Myc and NS4B-Flag were used as negative control. **(D)** GST pulldown assay. The GST or GST-Rab5 fusion proteins expressed in *E. coli* BL21 (DE3) were purified with glutathione agarose resin and incubated with the lysate of PK-15 cells overexpressing the Flag-tagged NS4B. After washing with cold PBS, the bound proteins were subjected to SDS-PAGE (12%) and western blot analysis using the anti-GST (PAb) (1:2000) and the anti-Flag MAb (1:1000).

Base on the findings that Rab5 coimmunoprecipitated with HCV NS4B protein (Stone et al., 2007), we hypothesized that Rab5 might also interact with CSFV NS4B protein. To substantiate this hypothesis, co-IP assays were performed to examine the possibility of Rab5 and NS4B forming a co-immunoprecipitable complex. The FLAG M2 Affinity Gel was applied to immunoprecipitate with the lysates from PK-15 cells transfected to express NS4B-Flag and western blot was carried out to analyze the proteins that bind to NS4B-Flag. As shown in **Figure 5A**, Rab5 was detected in the immunorecipitates using an anti-Rab5 antibody. Then, ectogenic NS4B-Flag and Rab5-Myc as well as NS5A-Flag and Rab5-Myc were co-expressed in PK-15 cells. As shown in **Figure 5B**, NS4B-Flag precipitated Rab5-Myc but the negative control NS5A-Flag could not. In the reciprocal co-IP assays, Rab2, which we previously showed to be associated with CSFV growth, served as a control (Liang et al., 2016). The reciprocal co-IP results indicated that Rab5-Myc also precipitated NS4B-Flag (**Figure 5C**) but Rab2-Myc did not, which is consistent with the results mentioned above (**Figures 5A,B**). Considering that CSFV proteins might be dysfunctional in some respects when expressed individually compared to expressed in the context of the viral polyprotein, PK-15 cells were infected with CSFV and co-transfected with pcDNA-NS4B-Flag and pcDNA-Rab5-Myc. The results obtained were similar to those of the reciprocal co-IP using anti-Myc antibody to precipitate NS4B-Flag (data not shown). These results demonstrate that the CSFV NS4B protein interacts with Rab5 protein in PK-15 cells.

To further confirm the interaction between CSFV NS4B and Rab5, we performed GST-pulldown assays. NS4B-Flag was expressed by HEK-293T cells transfected with pcDNA-NS4B-Flag. Rab5-GST and GST proteins were expressed and purified from bacteria transformed with pGEX-Rab5-GST and pGEX-6P-1, respectively. Rab5-GST or GST was fixed on glutathione agarose, and NS4B-Flag was added to the assay. Western blot was used to analyze proteins in the complexes using an anti-GST or anti-Flag antibody. **Figure 5D** showed that NS4B was detected in Rab5-GST complexes but not in GST complexes. These results further prove that NS4B does indeed interact with Rab5.

### CSFV NS4B Co-localizes with Rab5

**FIGURE 6:**
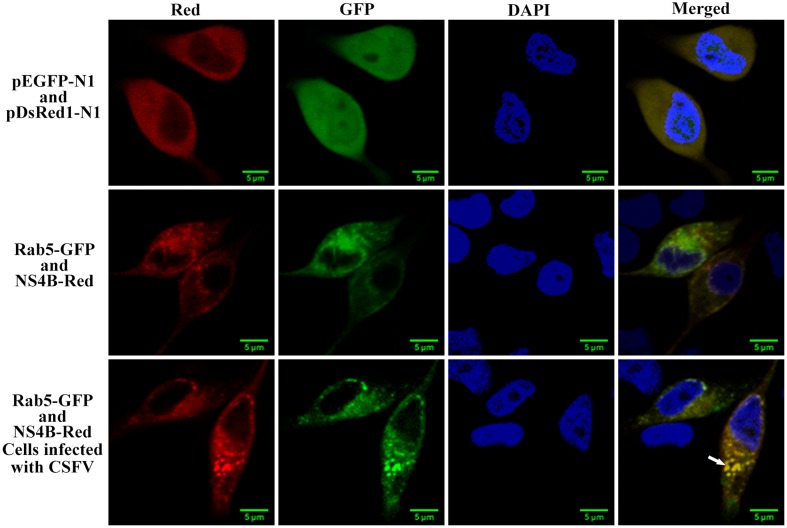
NS4B protein co-localizes with Rab5. PK-15 cells were co-transfected with pNS4B-Red and pRab5-GFP as well as pEGFP-N1 and pDsRed-N1 as control. Cells were fixed and stained with DAPI (blue) at 48 h post transfection. The NS4B-Red fusion protein was shown to co-localize with Rab5-GFP in both CSFV infected or uninfected cells. Scale bar = 5 μm.

Since we have demonstrated the direct interaction between Rab5 and CSFV NS4B, we want to know whether NS4B co-localize with Rab5. PK-15 cells co-expressing NS4B-Red and Rab5-GFP were analyzed under laser-scanning confocal microscopy. As shown in **Figure 6**, a co-localization of NS4B and Rab5 was observed in the cytoplasm of PK-15 cells. The co-localization of Rab5 and NS4B was more focused (white arrow) when cells were infected with CSFV. This might be a consequence of the polyprotein of CSFV. The co-localization coefficients were determined to be 0.81 and 0.78 in uninfected and CSFV infected cells, respectively. These results, in addition to previous results of co-IP and GST-pulldown assays, demonstrate that there was an interaction between CSFV NS4B and Rab5.

### Rab5 Facilitates the NS4B Related GFS Formation

**FIGURE 7:**
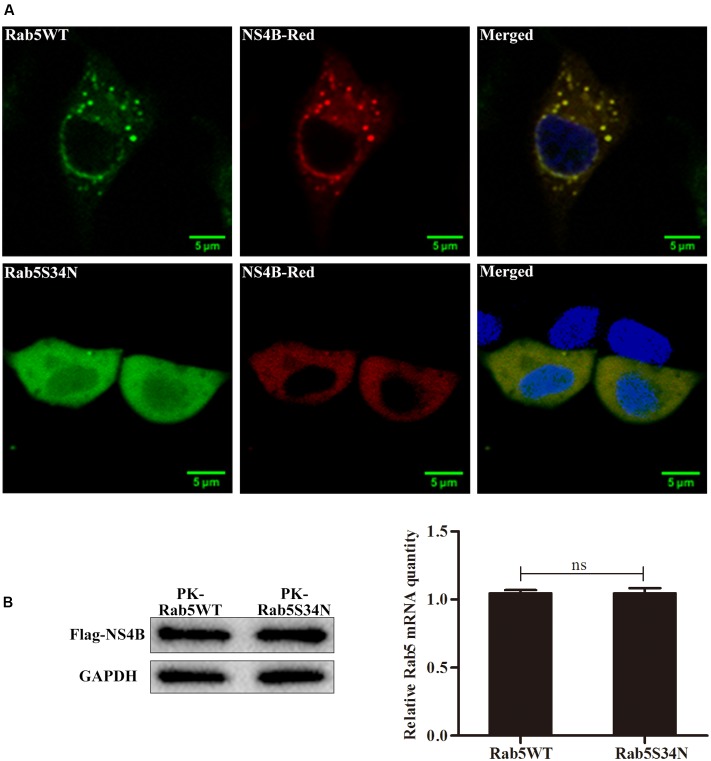
Rab5 facilitates the NS4B related GFS formation. PK-15 cells were infected with CSFV at an MOI of 1 and transfected or mock transfected with pRab5S34N-GFP 2 h post infection. The cells were cultured for 24 h and were transfected with pNS4B-Red. After another 48 h, the cells were fixed and stained with DAPI (blue). **(A)** NS4B-related GFS appeared in cells expressing Rab5WT but disappeared in cells expressing Rab5S34N. Scale bar = 5 μm. **(B)** The amounts of NS4B protein was accessed by western blot and presented no difference between Rab5WT and Rab5S34N expressing PK-15 cells. ^∗^*P* < 0.05; ^∗∗^*P* < 0.01; ^∗∗∗^*P* < 0.001; ns, no significant (*P* > 0.05).

To investigate the effect of Rab5 on the intracellular distribution of NS4B, a Rab5 dominant negative mutant Rab5S34N, which was shown to inhibit efficiently cellular basal Rab5 function, was used (Li and Stahl, 1993; Johns et al., 2009; Shi et al., 2016). PK-15 cells expressing Rab5S34N or Rab5WT were infected with CSFV and transfected with pFlagNS4B-Red. As shown in **Figure 7A**, NS4B distribution exhibited a distinct punctate pattern with numerous GFS in the cytoplasm of CSFV infected Rab5WT expressing PK-15 cells. However, the GFS formed by NS4B-Red disappeared in CSFV infected Rab5S34N expressing cells (**Figure 7A**). To clarify the reason of GFS disappearance, the expression level of NS4B was determined both in Rab5WT and Rab5S34N expressed cells. As shown in **Figure 7B**, no significant difference in the amounts of NS4B was found in both cells. These results indicate that NS4B was not degraded in Rab5S34N expressed cells. Taken together, these results show that functional Rab5 facilitates the formation of NS4B related GFS.

### NS4B Co-localizes with NS3 and NS5A in the GFS

**FIGURE 8:**
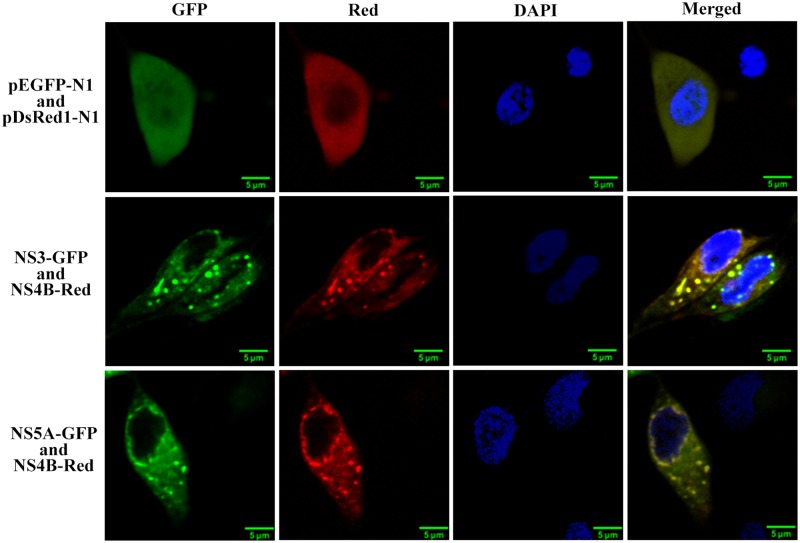
NS4B co-localizes with NS3 and NS5A proteins. pNS4B-Red together with pNS3-GFP or with pNS5A-GFP were transfected into CSFV-infected PK-15 cells. Cells were fixed and stained with DAPI (blue) at 48 h post transfection. The NS4B co-localized with NS3 and NS5A on GFS in a certain degree.

To investigate whether other viral replicase proteins were located in the GFS, pNS3-GFP or pNS5A-GFP was co-transfected with pFlagNS4B-Red into CSFV infected PK-15 cells. As shown in **Figure 8**, NS4B was shown to co-localize with NS3 or NS5A protein in the cytoplasm, especially in the NS4B related intracellular GFS. The co-localization coefficients for NS4B-NS3 and NS4B-NS5A were determined to be 0.89 and 0.83, respectively. These results suggest that both NS3 and NS5A were located in the NS4B related GFS.

## Discussion

Due to their limited genome, viruses employ host factors to complete their life cycles such as cell entry and gene synthesis. The small GTPase Rab5 is implicated in cell entry or/and multiplication of various viruses including adenovirus, HIV-1, dengue virus and West Nile virus (Rauma et al., 1999; Vidricaire and Tremblay, 2005; Krishnan et al., 2007). Rab5 has been shown to be required for cell entry of HCV and played a pivotal role in virus genome replication (Manna et al., 2010). Hepatitis B virus (HBV) infection strongly depends on Rab5 expression, but HBV replication is not affected by Rab5 downregulation (Macovei et al., 2013). Rab5 is also an important component during CSFV infection (Shi et al., 2016). However, how Rab5 affects CSFV proliferation remains unclear. In this study, we further demonstrated that Rab5 promotes CSFV propagation in PK-15 cells. CSFV growth was significantly enhanced by lentivirus-mediated constitutive and eukaryotic plasmid transient overexpression of Rab5 (**Figures 1**, **2**). Moreover, lentivirus-mediated shRNA knockdown of Rab5 drastically decreased CSFV production (**Figure 3**). These results suggested that Rab5 could positively promote CSFV propagation in PK-15 cells.

It is known that CSFV possesses the ability to modulate expression levels of host proteins that affect virus replication (Lin et al., 2014; Zhang et al., 2015; Ning et al., 2016). Caveolin-1 and heat shock protein 70 have been shown to promote CSFV propagation, and their genes expression levels were shown to be upregulated during CSFV proliferation (Zhang et al., 2015; Ning et al., 2016). Moreover, CSFV infection does not change the N^pro^-interacting protein of HS-1-associated protein X-1 (HAX-1) expression, which is known to promote virus replication (Johns et al., 2010). Our results showed that the expression levels of Rab5 were similar between CSFV infected and mock PK-15 cells at all examined time points (**Figure 4**). This result indicates CSFV did not modulate Rab5 expression level during the viral lifecycle.

Host factors affect virus replication usually via interacting with the viral genome or proteins. Eukaryotic elongation factor 1A binds to CSFV replicase protein NS5A to block virus replication (Li et al., 2015). Mitogen-activated protein kinase 2 interacts with CSFV main antigen protein E2 to promote CSFV growth via the attenuation of the JAK-STAT signaling pathway (Hu et al., 2016; Wang et al., 2016). Rab5 interacts with tomato bushy stunt virus replication protein to support virus replication and protects the virus from host antiviral responses (Xu and Nagy, 2016). Here, in addition to proving that Rab5 enhances CSFV growth, its interaction with viral NS4B protein was confirmed as well. The isolation of NS4B-Flag/Rab5 or Rab5-Myc/NS4B-Flag complexes (**Figures 5A–C**) suggested that Rab5 could bind to NS4B in PK-15 cells. GST-pulldown assays further demonstrates that Rab5 can bind to NS4B (**Figure 5D**) *in vitro*. Confocal microscopy showed the co-localization of Rab5 and NS4B, which confirmed the Rab5-NS4B interaction in PK-15 cells (**Figure 6**). Rab5 was found to co-immunoprecipitate with HCV NS4B protein and to play a role in NS4B-induced web formation related to HCV genome synthesis (Stone et al., 2007). Given the fact that CSFV and HCV have high similarity, it would be interesting to see whether CSFV NS4B induces a web formation and how the Rab5-NS4B interaction affects the NS4B-induced web formation.

Replication of all positive-strand RNA viruses cannot be separated from membrane-associated replication complexes (Miller and Krijnse-Locker, 2008). As a positive-stranded RNA virus, CSFV replication also requires intracellular membrane structures (Behrens et al., 1998; Wang et al., 1999). Our confocal microscopy assays illustrated that NS4B is distributed in the cytoplasm and that GFS appeared in PK-15 cells (**Figure 7A**). This GFS phenomenon caused by NS4B-Red was agreement with a previous finding that NS4B-GFP located in the cytoplasm with a tendency toward ‘dot-like’ granular structures (Tamura et al., 2015). Moreover, polymerized HCV NS4B has been shown to remodel host membranes to form replication complexes through binding to both viral replicase proteins and host cell factors like early endosome protein Rab5. These complexes appears as foci under fluorescence microscopy (Ishido et al., 1998; El-Hage and Luo, 2003; Yu et al., 2006; Manna et al., 2010). In the present study, we revealed that co-localization of NS4B with Rab5 exhibited a focus on GFS when cells were infected with CSFV (**Figure 6**), suggesting that the GFS were complexes formed by viral NS4B and cell factors including Rab5. We also observed that NS4B was co-localizing with viral NS3 and NS5A proteins, and that the co-localization also focused on the GFS in some degree (**Figure 8**), indicating that NS3 and NS5A might be components of a NS4B related complex. Rab5 was found in HCV NS4B-induced replication complexes and in replication compartments of tomato bushy stunt virus (Manna et al., 2010; Xu and Nagy, 2016). BVDV and HCV NS4B are known to associate with NS3 and NS5A to form virus replication complex (Qu et al., 2001; Aligo et al., 2009). CSFV NS3 and NS5A also possess indispensable features for virus propagation, such as helicase of NS3 and interaction of NS5A with virus 3′-untranslated region (Sheng et al., 2007, 2012; Wen et al., 2007). Therefore, we also speculate that the CSFV NS4B related complexes presenting as GFS in PK-15 cells might be viral replication complexes or harbored viral replication complexes. However, more studies need to be done to verify the exact content and subcellular localization of the complexes. NS4B distribution was more diffuse and GFS almost completely disappeared in PK-15 cells expressing Rab5S34N (**Figure 7A**). There are two situations that would lead to the disruption of GFS, (1) NS4B was degraded which would result in the disruption of the complexes. Alternatively, (2) the complexes were disbanded, but NS4B still exists. Our current results demonstrated that no significant difference were observed between the amounts of NS4B in Rab5WT and Rab5S34N expressing cells (**Figure 7B**), which indicates that NS4B related complexes were disbanded and that Rab5 facilitates NS4B related complexes formation.

In summary, this study demonstrates for the first time that the endosomal Rab5 small GTPase enhances CSFV growth and interacts with viral NS4B to facilitate the NS4B related complexes formation. Our study also reveals new information on CSFV-interacting host proteins and their impacts on CSFV replication. This will provide new opportunities to develop novel therapeutic approaches.

## Author Contributions

JL carried out all the experiments, collected data and wrote this manuscript. CW checked and revised the manuscript. LZ, TW, JZ, CL, GQ, and YO helped to finish the experiments. JL, CW, WL, KG, and YZ designed the experiments and analyzed the data. All authors discussed the results, commented on the manuscript and approved the final version.

## Conflict of Interest Statement

The authors declare that the research was conducted in the absence of any commercial or financial relationships that could be construed as a potential conflict of interest.
